# Prevalence of alcohol use disorders documented in electronic health records in primary care across intersections of race or ethnicity, sex, and socioeconomic status

**DOI:** 10.1186/s13722-024-00490-6

**Published:** 2024-08-30

**Authors:** Robert L. Ellis, Kevin A. Hallgren, Emily C. Williams, Joseph E. Glass, Isaac C. Rhew, Malia Oliver, Katharine A. Bradley

**Affiliations:** 1 Department of Health Systems and Population Health, University of Washington School of Public Health, Seattle, WA 98195 USA; 2 Kaiser Permanente Washington Health Research Institute, Seattle, WA 98101 USA; 3 Department of Psychiatry and Behavioral Sciences, University of Washington School of Medicine, Seattle, WA 98195 USA; 4 Health Services Research & Development (HSR&D) Center for Innovation for Veteran-Centered and Value-Driven Care, Veterans Affairs (VA) Puget Sound Health Care System, Seattle, WA 98101 USA; 5 Department of Medicine, University of Washington School of Medicine, Seattle, WA 98195 USA; 6 Center for Healthcare Policy and Research, University of California, Davis, 4900 Broadway Suite 1430, Sacramento, CA 95820 USA

**Keywords:** Alcohol, Race, SES, Sex, Primary care

## Abstract

**Background:**

Diagnosis of alcohol use disorder (AUD) in primary care is critical for increasing access to alcohol treatment. However, AUD is underdiagnosed and may be inequitably diagnosed due to societal structures that determine access to resources (e.g., structural racism that limits opportunities for some groups and influences interpersonal interactions in and beyond health care). This study described patterns of provider-documented AUD in primary care across intersections of race, ethnicity, sex, and community-level socioeconomic status (SES).

**Methods:**

This cross-sectional study used EHR data from a regional healthcare system with 35 primary care clinics that included adult patients who completed alcohol screenings between 3/1/2015 and 9/30/2020. The prevalence of provider-documented AUD in primary care based on International Classification of Diseases-9 (ICD-9) and ICD-10 diagnoses was compared across intersections of race, ethnicity, sex, and community-level SES.

**Results:**

Among 439,375 patients, 6.6% were Latine, 11.0% Asian, 5.4% Black, 1.3% Native Hawaiian/Pacific Islander (NH/PI), 1.5% American Indian/Alaska Native (AI/AN), and 74.2% White, and 58.3% women. The overall prevalence of provider-documented AUD was 1.0% and varied across intersecting identities. Among women, the prevalence was highest for AI/AN women with middle SES, 1.5% (95% CI 1.0–2.3), and lowest for Asian women with middle SES, 0.1% (95% CI 0.1–0.2). Among men, the prevalence was highest for AI/AN men with high and middle SES, 2.0% (95% CI 1.1–3.4) and 2.0% (95% CI 1.2–3.2), respectively, and lowest for Asian men with high SES, 0.5% (95% CI 0.3–0.7). Black and Latine patients tended to have a lower prevalence of AUD than White patients, across all intersections of sex and SES except for Black women with high SES. There were no consistent patterns of the prevalence of AUD diagnosis that emerged across SES.

**Conclusion:**

The prevalence of provider-documented AUD in primary care was highest in AI/AN men and women and lowest in Asian men and women. Findings of lower prevalence of provider-documented AUD in Black and Hispanic than White patients across most intersections of sex and SES differed from prior studies. Findings may suggest that differences in access to resources, which vary in effects across these identity characteristics and lived experiences, influence the diagnosis of AUD in clinical care.

**Supplementary Information:**

The online version contains supplementary material available at 10.1186/s13722-024-00490-6.

## Background

Alcohol use disorder (AUD) is a common, debilitating, and deadly condition [49, 53] that affects over 14.5 million adults living in the United States [56]. Furthermore, AUD prevalence is increasing among vulnerable populations, including minoritized racial and ethnic groups, women, and persons with lower education and socio-economic status [23, 24, 54]. Diagnosing AUD in primary care settings is a critical step in increasing access to available AUD treatments and interventions. However, AUD is often underdiagnosed and/or inequitably diagnosed, in the U.S. general population and clinical care settings, including primary care, [8, 35], potentially resulting in millions of Americans not receiving care for this treatable health condition.

Fundamental Cause Theory suggests that health and healthcare outcomes (e.g., AUD and access to diagnosis and treatment for diseases like AUD) and disparities therein are influenced by upstream social factors that determine access to resources (e.g., education, occupation, income, and other markers of socioeconomic status; neighborhood opportunities). These upstream factors are socially patterned in ways that privilege some persons and disadvantage others. For instance, structural racism is a fundamental cause that has determined access to resources for centuries in differing ways over time, despite interventions (e.g., abolition of slavery), and has severely limited opportunities and outcomes, including health and health outcomes, for Black and other minoritized Americans. Similarly, sexism has created structural barriers for women seeking care for a stigmatized condition such as AUD. Women experience more logistical challenges as primary caregivers, higher healthcare costs despite earning less than men, and a greater amount of stigma for using alcohol [9, 52, 60]. Additionally, individuals with lower SES experience poorer health outcomes and have less access to medical care, including care for AUD [5, 41]. Fundamental Cause Theory suggests that contextualizing individually-based risk factors (e.g., race) within structural determinants of access to resources (e.g., racism) is necessary to create effective interventions that address root causes of poor and disparate health outcomes [43].

Public Health Critical Race Praxis (PHCRP), an analytical framework integrating critical race theory with public health research and practice [17], calls for researchers to contextualize individual differences by focusing on power hierarchies underlying access to resources. PHCRP also expands this to focus on intersectionality, or how interlocking systems of power (e.g., racism, sexism, and elitism) manifest to uniquely and differentially shape the lived experiences of persons with intersectional social positions or identities (e.g., race, gender, and class) [10]. Taken together, race, sex, and SES have been the most influential factors used to organize people worldwide [2] and significantly impact access to healthcare resources in American society [39, 44].

Prior studies have described the prevalence and consequences of AUD across subgroups based on individual identity characteristics. These studies have shown that the prevalence of AUD varies across subgroups in the U.S. general population, with the highest prevalence among American Indian and Alaska Native (AI/AN) individuals, non-Latine White individuals, and men [23, 24, 54], but with minority racialized groups, women, and individuals with lower socioeconomic status (SES) experiencing greater social and medical consequences associated with AUD [11, 37, 46, 47]. Further, persons with multiple overlapping identities may experience multiplicative risks associated with AUD [12, 20].

Prior studies in the Veteran Health Administration (VA) have highlighted potential inequalities across race, ethnicity, and sex in AUD diagnosis documented by providers in electronic health records (EHRs) in clinical settings [64, 66, 67]. In a study of VA patients nationwide, Black and Latine patients had a higher prevalence of documented AUD than White patients [67]. In contrast, in population-based interview studies of the US population, White patients have a higher AUD prevalence (Bridget F. [23, 24]). Moreover, the prevalence of AUD in an adult general US population sample compared to VA (11.3% vs. 6.5%, respectively), suggests underdiagnoses associated with documenting AUD based on provider discretion [56, 67]. Another study found similar findings even when stratified by level of alcohol consumption based on alcohol screening scores [64]. Finally, a study comparing EHR-documented AUD in the VA to diagnostic interviews for AUD found that AUD was under-diagnosed across all sex and racial groups. However, these findings may not generalize to non-VA settings. Moreover, these prior studies have focused on individual identity characteristics instead of on the power structures that influence their outcomes and have not focused on understanding differences in AUD from a structural perspective (i.e., through the lenses of Fundamental Cause Theory and the PHCRP) and have not assessed lived experiences that result from structures (e.g., neighborhood socioeconomic status). Studying AUD through these lenses might help clinicians and researchers understand patterns in novel ways that could support better intervention development to increase equity in access to diagnosis and treatment. Describing patterns of health outcomes across subgroups based on the intersection of identities and lived experiences is often a first step in understanding intersectional oppression.

This study aimed to describe patterns in the prevalence of provider-documented AUD in primary care across intersections of race or ethnicity, sex, and SES (“race, ethnicity, sex, and SES” hereafter) through a structural lens. Complementing prior work conducted within the VA [64, 67], in addition to adding intersectionality with SES, the present study was conducted in a large regional health system that has a greater representation of women and people of all ages, facilitating comparisons with the US general population.

## Methods

### Study design and data source

**Fig. 1:**
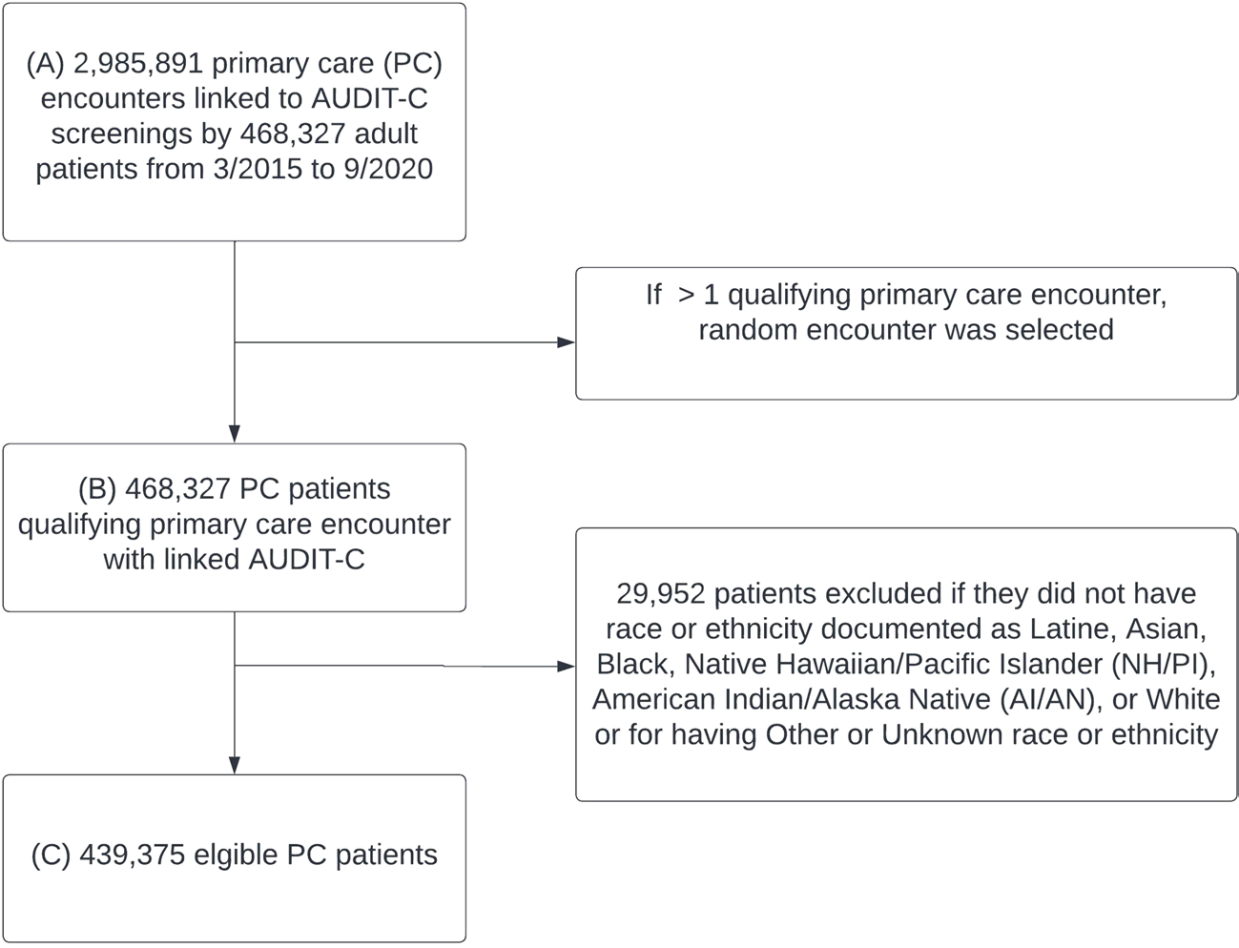
Study sample of primary care patients: inclusion and exclusions

This cross-sectional study was conducted in Kaiser Permanente Washington (KPWA), an integrated health system in Washington State. Patients were eligible if they: (1) were ≥ 18 years old, (2) had ≥ 1 primary care encounter in one of 35 KPWA primary care clinics 03/2015–09/2020, and (3) had alcohol screening documented in the EHR within the prior year. If more than one encounter was eligible for a patient, one was randomly selected as the index encounter. Patients were excluded if they did not have race or ethnicity documented as Latine, Asian, Black, Native Hawaiian/Pacific Islander (NH/PI), American Indian/Alaska Native (AI/AN), or White, or if their race or ethnicity were documented as “other” or “unknown” (Fig. 1). This study was approved by KPWA Health Research Institute’s Review Board with a waiver of consent and HIPAA authorization to use existing EHR data for research.

### Measures

#### Predictor variables of interest

##### Race or ethnicity

Race and ethnicity were extracted from the EHR and categorized into 6 mutually exclusive groups (Latine, non-Latine Asian, non-Latine Black, non-Latine NH/PI, non-Latine AI/AN, and non-Latine White). KPWA collected data on race and ethnicity for the EHR using a voluntary demographic questionnaire sent by email [34]; when patients did not complete the questionnaire, they were asked for this information at their next visit. Each member could be classified in one of two ethnicity categories (Latine or non-Latine) and up to 7 racial categories (Asian, Black, Native Hawaiian/Pacific Islander (NH/PI), American Indian/Alaska Native (AI/AN), White, Other, and Unknown). Patients who reported multiple races (other than Latine) were assigned to their reported racial group with the smallest count within the study sample, consistent with other epidemiological studies [23, 24, 50, 51].

##### Sex

Sex (male/man or female/woman) was extracted from the EHR and could represent either sex or gender.

##### Community-level Socioeconomic status (SES)

Messer’s Neighborhood Deprivation Index (MNDI) was used to estimate patients’ community-level SES (SES hereafter). The MNDI is a validated index [14, 40, 57] providing census tract-level measures of SES based on measures of education, housing, income, employment, public assistance, and family structure from the American Community Survey [45]. Principle component analysis (PCA) was used to standardize MDNI scores and to determine the weight of each variable [45] (Supplemental Table 1). For the current study, the MNDI was obtained based on patients' residential addresses recorded during the index visit and scores were categorized into low, middle, or high terciles of SES based on whether patients were in the lower, middle, or top third of the distribution of MNDI scores in the study sample.

### Intersectionality of race, ethnicity, sex, and SES

Race, ethnicity, sex, and SES were combined to generate composite race, ethnicity and terciles of SES variables of eighteen intersectional identities for women and men separately (36 total intersectional identities). For example, Black women with low SES or Asian men with high SES. These composite variables reflect intersecting identities created by systems of power and oppression [7, 17, 18, 25] and represent the interlocking and involuntary experiences that individuals with multiple oppressions (racism, sexism, social classism) face as they attempt to gain access or interface with healthcare, which in turn may impact the likelihood of being diagnosed and treated with AUD. For instance, Black women from lower SES have historically and currently experienced different levels of access to healthcare services compared to White women from lower SES, who, in turn, have vastly different levels of access than White women from higher SES [13].

Intersections of EHR-documented race, ethnicity, sex, and SES were used as proxies for upstream factors of racism, sexism, and social classism, as they are routinely collected in EHRs, and were combined with census data to estimate community-level SES. Although EHR data does not directly capture racism, sexism, and social classism, these proxies allow us to assess how societal structures might impact diagnosis patterns of AUD in real-world clinical settings, including primary care, which are often affected by implicit bias and a history of discrimination. Primary care is an ideal setting for diagnosing and treating AUD, as most adults (80%) access primary care services and can receive effective treatments, such as medications for AUD [48].

#### Primary outcome

*Provider-documented AUD* was defined as the presence of any active AUD diagnosis documented by a provider in the EHR within primary care on the day of an index primary care encounter or in the following 365 days. Active AUD diagnoses were defined based on the International Classification of Diseases 9th and 10th Editions (ICD-9 and ICD-10; Supplemental Table 2) [64, 67]. Diagnoses from KPWA insurance claims were not included in this study to allow a study of diagnoses made by providers who could view routine alcohol screening results.

#### Covariates

*Age* (continuous) at index visit was extracted from EHRs. *Days enrolled* in the KPWA system in the two years prior to the index visit and days enrolled in the KPWA system over the one year after the index visit were included to account for eligibility for KPWA care. *Clinics* where index visits occurred and *calendar years* of the visits were included to account for potential differences in AUD diagnosing practices across clinics and changes over time, respectively.

*Alcohol use* was measured with the Alcohol Use Disorders Identification Test-Consumption (AUDIT-C) questionnaire (Supplemental Fig. 2) [3, 19, 33]. The most recent AUDIT-C score (0–12) prior to the index visit was used to adjust for differences in alcohol use across intersections of race, ethnicity, sex, and SES and increased likelihood of receiving AUD diagnosis for higher levels of alcohol use [64, 66]. All study clinics offered annual alcohol screening [42] during the study period, with 91% screened in February 2020 [27]. The EHR automatically prompted medical assistants to ask patients to complete screening on paper if not completed within the past year; medical assistants aimed to enter responses into the EHR before visits. For descriptive purposes, AUDIT-C scores (0–12) are categorized into 5 risk levels: no drinking (score of 0 points), low-level drinking (1–2 women/1–3 men), mild (3–6 women/4–6 men), moderate (7 to 8), and severe (9–12) unhealthy alcohol use.

Previous *EHR-documented alcohol and substance use disorders*, *medical conditions* that are highly attributable to alcohol (e.g., liver disease, pancreatitis, and cirrhosis), and *mental health diagnoses* (e.g., depression, anxiety, and bipolar disorder) required one relevant inpatient or two relevant outpatient ICD-9 or ICD-10 diagnostic codes in the EHR within two years prior to the index visit (Supplemental Table 3) [31].

### Analyses

#### Descriptive statistics

We described demographic and clinical characteristics overall and across racial and ethnic subgroups within women and men, separately, to account for sex-related differences in the prevalence of AUD [16]. Within these subgroups, we used the chi-square test of independence to compare the proportions of terciles of SES (low SES, middle SES, and High SES), insurance types, days of enrollment in the KPWA system, previous alcohol and substance use, medical conditions attributable to alcohol use, mental health diagnosis, and alcohol use.

#### Primary analyses

Generalized linear models (GLMs) with marginal standardization were performed, in a stepped manner to incrementally adjust for confounders, to estimate predicted prevalence estimates of provider-documented AUD for each of the categorized intersectional identities. This method was chosen over other intersectional analytic methods because our main objective was to describe patterns of AUD across investigator-created intersectional identities. Further, this method eliminated the need for a comparator group, another recommendation PHCRP. Lastly, this method effectively translates real-world data from EHRs into usable and easy-to-understand results (i.e., percentages of patients in each subgroup with provider-documented AUD), which may aid interpretation and translation to real-world care settings.

The predicted prevalence and 95% confidence intervals (CIs) of provider-documented AUD in primary care were estimated for each of the 18 subgroups reflecting the intersection of race or ethnicity, and tercile of SES, with separate models for women and men. Model 1 was unadjusted. Model 2 adjusted for *age*, *days enrolled*, *calendar year*, and *clinic*. Model 3 added adjustment for AUDIT-C score (0–12) to adjust for past-year alcohol consumption. Model 4 added adjustments for *EHR-documented diagnoses of alcohol and substance use disorders*, *medical conditions* attributable to alcohol, and *mental health diagnoses in the past 2 years*. These models were specified a priori to determine whether patterns of clinical diagnosis changed when adjusting for different measures that could affect diagnosing practices. Additionally, a sensitivity analysis was performed that repeated Models 1–3 on a sample restricted to patients without any alcohol and substance use disorder diagnoses documented in the prior 2 years.

Prior to modeling, we conducted power analyses to estimate the precision of estimates based on anticipated 95% confidence limits (CIs), assuming an AUD prevalence rate of 5% [67] within the estimated size of the smallest minoritized subgroup (N = 527, expected 95%:3.4–7.3) and largest minoritized group (N = 10,798, expected 95% CI:4.6–5.4), which we judged to be an adequate level of precision. All analyses were performed using R Version 4.2.0.

## Results

### Demographic and clinical characteristics

Among 439,375 eligible primary care patients included in this study, 59% (N = 259,008) were women (Table 1a), and 41% (N = 180,367) were men (Table 1b). Among women, 7.0% were Latine, 11.7% Asian, 5.4% Black, 1.3% NH/PI, 1.6% AI/AN, and 73.0% White. Among men, 6.0% were Latine, 10.0% Asian, 5.5% Black, 1.3% NH/PI, 1.3% AI/AN, and 75.9% White. Among both women and men, Latine, Black, NH/PI, and AI/AN patients had an elevated probability of being in the lowest tercile of community SES (40.1–52.9%) and lowest probability of being in the highest tercile of community SES (16.5–28.1%). Women were younger and had a higher proportion of Medicaid insurance compared to men of all racial or ethnic subgroups. Latine, Black, and NH/PI patients tended to be younger than White patients, who had the highest proportion of patients insured through Medicare. Commercial or private insurance was the most common among all racial or ethnic groups for both women and men. Low-level drinking was the most common drinking category across all racial or ethnic groups except for Asian women, for whom non-drinking was most common. Patients with moderate to severe unhealthy alcohol use made up a small proportion of patients, but the proportion was higher in men than women.

**Table 1:** **a.** Demographic and Clinical Characteristics of Women in Primary Care Across Race or Ethnicity. **b.** Demographic and Clinical Characteristics of Men in Primary Care Across Race or Ethnicity

a							
Women N = 259,008	Latine N = 18,120	Asian N = 30,331	Black N = 13,929	NH/PI N = 3,496	AI/AN N = 4046	White N = 189,086	P
Age (y) – Mean (SD)	42.9 (17.1)	45.2 (16.3)	43.3 (16.6)	40.2 (15.0)	47.5 (17.4)	50.7 (18.5)	
Terciles of Socioeconomic Status (SES), %							< 0.001
Lowest SES	40.1	31.5	52.9	45.2	44.2	32.3	
Middle SES	31.7	31.9	30.6	36.9	33.7	33.7	
Highest SES	28.2	36.6	16.5	17.9	22.1	34.0	
Insurance Type, %							< 0.001
Medicaid	4.2	3.0	7.7	5.2	5.9	3.1	
Medicare	12.5	11.5	12.0	7.1	19.0	24.2	
Commercial or Private	73.5	75.2	72.8	78.2	64.8	61.0	
State subsidized	4.1	7.0	2.3	3.5	5.1	4.7	
Other or Unknown	5.7	3.3	5.2	6.0	5.2	7.0	
Mean Percentage of Days of Enrolled in KPWA within 2 Years Pre-Index Visit and 1 Year Post-Index Visit, %							
Pre-Index Visit	69.4	71.0	72.6	69.4	75.3	74.1	< 0.001
Post-Index Visit	79.3	83.0	80.7	79.1	80.2	81.8	< 0.001
Alcohol and Substance Use Disorder Diagnoses in Prior 2-years, %							
Any Prior Diagnoses	1.3	0.4	1.6	0.9	3.0	2.0	< 0.001
Alcohol	0.8	0.2	0.8	0.5	1.6	1.2	< 0.001
Opioid	0.3	0.1	0.4	0.2	0.9	0.5	< 0.001
Stimulant	0.1	0.0	0.1	0.0	0.2	0.1	= 0.006
Cannabis	0.2	0.1	0.3	0.1	0.4	0.2	< 0.001
Other Drugs	0.2	0.0	0.2	0.1	0.4	0.2	< 0.001
Any Alcohol-Attributable Conditions in Prior 2-year, %							
Any Alcohol Condition	1.9	1.3	1.2	1.3	1.9	1.5	< 0.001
Liver Disease^a^	1.6	1.2	1.0	1.0	1.4	1.2	< 0.001
Pancreatitis^b^	0.3	0.1	0.3	0.3	0.5	0.3	< 0.001
Other Conditions^c^	0.0	0.0	0.0	0.0	0.1	0.1	= 0.005
Mental Health Diagnoses in Prior 2-year, %							
Any Mental Diagnoses	22.1	10.6	19.6	15.3	31.6	27.9	< 0.001
Depression	14.5	6.6	12.8	9.7	21.0	18.1	< 0.001
Anxiety	13.4	6.1	11.4	8.8	19.1	16.1	< 0.001
Bipolar	1.3	0.5	1.3	1.1	2.2	1.8	< 0.001
ADHD	1.5	0.7	1.3	1.1	2.2	1.8	< 0.001
PTSD	1.5	0.4	1.5	0.9	3.2	1.4	< 0.001
Schizophrenia	0.2	0.1	0.3	0.2	0.2	0.1	< 0.001
Eating Disorder	0.3	0.1	0.2	0.2	0.4	0.3	< 0.001
Other Psychosis	0.1	0.1	0.2	0.1	0.1	0.1	= 0.701
AUDIT-C Risk Levels – Score Range (0–12), %							< 0.001
No drinking (0)	31.9	50.7	39.7	34.7	35.3	27.2	
Low-level drink (1–2)	44.3	35.0	40.3	41.0	40.9	41.6	
Mild UAU (3–6)	22.9	13.9	18.9	23.2	22.4	30.3	
Moderate UAU (7–8)	0.6	0.3	0.8	0.9	1.0	0.6	
Severe UAU (9–12)	0.3	0.1	0.3	0.2	0.4	0.3	

b							
Men N = 180,367	Latine N = 10,812	Asian N = 18,022	Black N = 9,856	NH/PI N = 2,432	AI/AN N = 2393	White N = 136,852	P
Age (y) – Mean (SD)	41.8 (15.7)	46.0 (16.7)	45.0 (16.2)	42.3 (14.9)	48.9 (17.9)	51.7 (18.1)	
Terciles of Socioeconomic Status (SES), %							< 0.001
Lowest SES	39.3	30.1	49.1	43.3	40.7	30.1	
Middle SES	31.9	31.7	32.3	36.6	33.8	33.9	
Highest SES	28.8	38.2	18.6	20.1	25.5	36.0	
Insurance Type, %							< 0.001
Medicaid	2.8	2.4	4.4	2.7	4.0	2.0	
Medicare	9.7	13.3	12.4	9.0	23.2	26.1	
Commercial or Private	77.8	73.9	75.5	79.4	63.7	60.9	
State subsidized	3.7	7.2	2.4	3.6	4.3	4.4	
Other or Unknown	6.0	3.2	5.3	5.3	4.8	6.5	
Mean Percentage of Days of Enrolled in KPWA within 2 Years Pre-Index Visit and 1 Year Post-Index Visit, %							
Pre-Index Visit	65.7	70.9	71.7	71.6	75.8	74.5	< 0.001
Post-Index Visit	79.1	83.0	80.8	81.3	82.0	82.4	< 0.001
Alcohol and Substance Use Disorder Diagnoses in Prior 2-years, %							
Any Prior Diagnoses	2.3	0.8	2.4	1.6	3.8	3.0	< 0.001
Alcohol	1.5	0.5	1.5	0.6	2.3	2.1	< 0.001
Opioid	0.4	0.2	0.4	0.6	0.9	0.5	< 0.001
Stimulant	0.2	0.1	0.2	0.3	0.3	0.1	< 0.001
Cannabis	0.4	0.1	0.4	0.4	0.4	0.3	< 0.001
Other Drugs	0.2	0.1	0.2	0.2	0.5	0.2	< 0.001
Any Alcohol-Attributable Conditions in Prior 2-year, %							
Any Alcohol Condition	2.1	2.0	1.6	2.1	3.0	1.8	< 0.001
Liver Disease^a^	1.8	1.8	1.1	1.7	2.7	1.4	< 0.001
Pancreatitis^b^	0.3	0.2	0.4	0.3	0.4	0.3	= 0.005
Other Conditions^c^	0.1	0.0	0.1	0.1	0.1	0.1	= 0.005
Mental Health Diagnoses in Prior 2-year, %							
Any Mental Diagnoses	13.6	7.3	11.1	10.4	19.3	17.2	< 0.001
Depression	7.7	4.2	6.4	5.7	11.8	10.1	< 0.001
Anxiety	7.7	3.9	5.5	5.6	10.3	9.1	< 0.001
Bipolar	0.7	0.3	0.7	0.7	1.5	1.1	< 0.001
ADHD	1.9	0.9	1.4	1.5	2.2	2.1	< 0.001
PTSD	0.7	0.2	0.6	0.7	1.6	0.6	< 0.001
Schizophrenia	0.2	0.1	0.4	0.3	0.3	0.2	< 0.001
Eating Disorder	0.1	0.0	0.0	0.0	0.0	0.0	= 0.333
Other Psychosis	0.1	0.1	0.3	0.1	0.1	0.1	= 0.002
AUDIT-C Risk Levels – Score Range (0–12), %							< 0.001
No drinking (0)	24.0	33.5	34.6	28.7	33.4	24.4	
Low-level drink (1–3)	48.4	48.7	46.3	46.2	43.1	46.4	
Mild UAU (4–6)	23.8	16.5	16.5	21.6	20.3	26.0	
Moderate UAU (7–8)	2.6	1.3	1.7	2.4	2.0	2.2	
Severe UAU (9–12)	1.2	0.5	0.9	1.1	1.2	1.0	

^a^Liver Disease includes liver disease, liver cirrhosis, portal hypertension, and esophageal varices. ^b^Pancreatitis includes acute, alcohol-induced acute, chronic, and alcohol-induced chronic pancreatitis. ^c^Other Conditions include alcohol polyneuropathy, cardiomyopathy, alcoholic gastritis, alcoholic psychosis, generation of the nervous system due to alcohol, gastroesophageal hemorrhage, and alcoholic myopathy. UAU = unhealthy alcohol use

### Unadjusted prevalence of provider-documented AUD in primary care

The unadjusted prevalence of provider-documented AUD in the overall sample was 1.0% and varied across intersections of race, ethnicity, sex, and SES (Table 2: Model 1). Results show inconsistent patterns across low, middle, and high terciles of SES across sex and race or ethnic groups (Table 2: Model 1). Asian patients had the lowest prevalence of AUD compared to other races or ethnicities. Men tended to have a higher prevalence compared to women. Among men, the prevalence of AUD was highest for AI/AN men with high and middle SES, 2.0% (95% CI 1.1–3.4) and 2.0% (95% CI 1.2–3.2), respectively, and lowest among Asian men with high SES, 0.5% (95% CI 0.3–0.7) (Table 2: Model 1). Among women, the prevalence of AUD was highest among AI/AN women with middle SES, 1.5% (95% CI 1.0–2.3), and lowest among Asian women with middle SES, 0.1% (95% CI 0.1–0.2) (Table 2: Model 1). Of note, point estimates of the prevalence of provider-documented AUD in White patients were higher than in Black or Latine patients across all intersections of sex and SES terciles, except Black women with high SES.

**Table 2:** Prevalence of Provider-Documented Alcohol Use Disorders (95% Confidence Intervals) among Primary Care Patients Across Intersections of Race, Ethnicity, and Terciles of SES, Stratified by Sex

	Women			Men		
	Low SES N = 88,268	Mid SES N = 85,660	High SES N = 83,980	Low SES N = 57,559	Mid SES N = 60,167	High SES N = 61,847
Model 1 (Main Model): Unadjusted						
Latine	0.6 (0.4–0.8)	0.5 (0.4–0.7)	0.6 (0.4–0.8)	1.4 (1.1–1.8)	1.4 (1.1–1.8)	1.3 (0.9–1.8)
Asian	0.2 (0.1–0.3)	0.1 (0.1–0.2)	0.2 (0.1–0.3)	0.6 (0.4–0.9)	0.8 (0.6–1.1)	0.5 (0.3–0.7)
Black	0.7 (0.5–0.9)	0.6 (0.4–0.9)	0.9 (0.6–1.3)	1.6 (1.3–2.0)	1.2 (0.9–1.6)	1.1 (0.7–1.7)
NH/PI	0.4 (0.2–0.8)	0.3 (0.1–0.8)	0.2 (0.0–1.1)	1.4 (0.9–2.4)	1.0 (0.5–1.9)	1.6 (0.8–3.2)
AI/AN	0.6 (0.3–1.1)	1.5 (1.0–2.3)	0.8 (0.4–1.6)	1.5 (0.9–2.5)	2.0 (1.2–3.2)	2.0 (1.1–3.4)
White	0.8 (0.8–0.9)	0.7 (0.7–0.8)	0.7 (0.7–0.8)	1.8 (1.7–2.0)	1.7 (1.6–1.8)	1.5 (1.4–1.6)
Model 2: Additionally adjusts for age, days enrolled, year of the index visit, and clinic						
Latine	0.5 (0.4–0.7)	0.5 (0.3–0.7)	0.5 (0.4–0.8)	1.3 (1.0–1.6)	1.3 (1.0–1.7)	1.1 (0.8–1.6)
Asian	0.2 (0.1–0.3)	0.1 (0.1–0.2)	0.2 (0.1–0.3)	0.6 (0.4–0.8)	0.8 (0.6–1.0)	0.5 (0.3–0.7)
Black	0.6 (0.4–0.8)	0.6 (0.4–0.8)	0.8 (0.5–1.2)	1.5 (1.2–1.8)	1.1 (0.8–1.5)	1.0 (0.6–1.5)
NH/PI	0.3 (0.2–0.8)	0.3 (0.1–0.8)	0.1 (0.0–1.1)	1.3 (0.8–2.2)	0.9 (0.5–1.8)	1.5 (0.8–3.0)
AI/AN	0.6 (0.3–1.0)	1.3 (0.8–2.0)	0.7 (0.3–1.5)	1.4 (0.9–2.3)	1.8 (1.1–2.9)	1.8 (1.0–3.2)
White	0.8 (0.7–0.9)	0.7 (0.6–0.8)	0.7 (0.6–0.8)	1.8 (1.6–1.9)	1.6 (1.5–1.7)	1.4 (1.3–1.5)
Model 3: Additionally adjusts for AUDIT-C (0–12)						
Latine	0.3 (0.2–0.4)	0.3 (0.2–0.4)	0.3 (0.2–0.4)	0.8 (0.6–1.1)	0.8 (0.6–1.0)	0.7 (0.5–1.0)
Asian	0.2 (0.1–0.3)	0.2 (0.1–0.3)	0.1 (0.1–0.2)	0.5 (0.3–0.7)	0.6 (0.5–0.8)	0.4 (0.3–0.5)
Black	0.4 (0.3–0.5)	0.3 (0.2–0.5)	0.4 (0.3–0.7)	1.0 (0.8–1.3)	0.9 (0.6–1.3)	0.7 (0.4–1.1)
NH/PI	0.2 (0.1–0.4)	0.2 (0.1–0.5)	0.1 (0.0–0.6)	0.8 (0.5–1.4)	0.6 (0.3–1.1)	1.1 (0.5–2.2)
AI/AN	0.3 (0.2–0.6)	0.6 (0.4–1.0)	0.4 (0.2–0.8)	0.9 (0.5–1.5)	1.2 (0.7–2.0)	1.0 (0.6–1.9)
White	0.4 (0.3–0.5)	0.3 (0.3–0.4)	0.3 (0.3–0.4)	1.0 (0.9–1.1)	1.0 (0.9–1.1)	0.8 (0.8–0.9)
Model 4: Additionally adjusts for alcohol and substance use disorders, alcohol-attributable conditions, and mental health (diagnoses from past 2 years of index visit)						
Latine	0.2 (0.2–0.3)	0.2 (0.1–0.3)	0.3 (0.2–0.4)	0.7 (0.5–1.0)	0.6 (0.5–0.9)	0.5 (0.4–0.7)
Asian	0.2 (0.1–0.3)	0.1 (0.0–0.2)	0.1 (0.1–0.2)	0.4 (0.3–0.6)	0.6 (0.4–0.8)	0.3 (0.2–0.5)
Black	0.3 (0.2–0.4)	0.3 (0.2–0.4)	0.4 (0.2–0.6)	0.8 (0.6–1.1)	0.8 (0.5–1.1)	0.6 (0.4–1.0)
NH/PI	0.2 (0.1–0.4)	0.2 (0.1–0.4)	0.1 (0.0–0.7)	0.7 (0.4–1.3)	0.6 (0.3–1.1)	1.0 (0.5–2.1)
AI/AN	0.2 (0.1–0.4)	0.5 (0.3–0.8)	0.3 (0.1–0.7)	0.8 (0.5–1.4)	1.0 (0.6–1.7)	0.9 (0.5–1.7)
White	0.3 (0.3–0.3)	0.3 (0.2–0.3)	0.2 (0.2–0.3)	0.8 (0.7–0.9)	0.8 (0.7–0.9)	0.7 (0.6–0.8)

### Adjusted prevalence of provider-documented AUD

After adjusting for age, days enrolled, calendar year, and clinic, variation was consistent with patterns observed in the unadjusted model. Among men, the highest prevalence remained among AI/AN men with middle and high SES, and lowest among Asian men with high SES (Table 2: Model 2). Among women, the highest prevalence remained among AI/AN women with middle SES and the lowest among Asian women with middle SES, although NH/PI women with high SES were now comparably low (Table 2: Model 2).

After additionally adjusting for AUDIT-C scores, observable patterns of the prevalence of AUD continued to vary across race or ethnicity, sex, and terciles of SES (Table 2: Model 3). However, differences in AUD prevalence across race, ethnicity, and sex were attenuated.

Adding adjustments for past alcohol and substance use disorders, alcohol-associated medical conditions, and mental health diagnoses, did not meaningfully change observable patterns (Table 2: Model 4).

Sensitivity analyses in a sample restricted by removing 9,252 patients with prior alcohol and substance use disorders, showed similar patterns as previous models (Table 3).

**Table 3:** Sensitivity Analysis of Prevalence of Provider-Documented Alcohol Use Disorders (95% Confidence Intervals) among a Restricted Primary Care Patients Sample with No Prior Alcohol or Substance Use Disorder Across Intersections of Race and Ethnicity, and Terciles of SES, Stratified by Sex

	Women			Men		
	Low SES N = 86,542	Mid SES N = 84,181	High SES N = 82,772	Low SES N = 55,846	Mid SES N = 58,548	High SES N = 60,376
Model 1: Unadjusted						
Latine	0.6 (0.4–0.8)	0.5 (0.4–0.7)	0.6 (0.4–0.8)	1.4 (1.1–1.8)	1.4 (1.1–1.8)	1.3 (0.9–1.8)
Asian	0.2 (0.1–0.3)	0.1 (0.1–0.2)	0.2 (0.1–0.3)	0.6 (0.4–0.9)	0.8 (0.6–1.1)	0.5 (0.3–0.7)
Black	0.7 (0.5–0.9)	0.6 (0.4–0.9)	0.9 (0.6–1.3)	1.6 (1.3–2.0)	1.2 (0.9–1.6)	1.1 (0.7–1.7)
NH/PI	0.4 (0.2–0.8)	0.3 (0.1–0.8)	0.2 (0.0–1.1)	1.4 (0.9–2.4)	1.0 (0.5–1.9)	1.6 (0.8–3.2)
AI/AN	0.6 (0.3–1.1)	1.5 (1.0–2.3)	0.8 (0.4–1.6)	1.5 (0.9–2.5)	2.0 (1.2–3.2)	2.0 (1.1–3.4)
White	0.8 (0.8–0.9)	0.7 (0.7–0.8)	0.7 (0.7–0.8)	1.8 (1.7–2.0)	1.7 (1.6–1.8)	1.5 (1.4–1.6)
Model 2: Additionally adjusts for age, days enrolled, year of the index visit, and clinic						
Latine	0.3 (0.2–0.4)	0.3 (0.2–0.5)	0.4 (0.2–0.6)	0.9 (0.7–1.2)	0.8 (0.6–1.2)	0.7 (0.5–1.0)
Asian	0.1 (0.1–0.2)	0.0 (0.0–0.1)	0.1 (0.1–0.2)	0.4 (0.2–0.5)	0.5 (0.4–0.7)	0.3 (0.2–0.5)
Black	0.3 (0.2–0.5)	0.3 (0.2–0.5)	0.5 (0.3–0.9)	0.9 (0.7–1.2)	0.8 (0.6–1.2)	0.8 (0.5–1.3)
NH/PI	0.3 (0.1–0.6)	0.1 (0.0–0.5)	0.1 (0.0–1.0)	0.8 (0.4–1.5)	0.7 (0.3–1.5)	1.4 (0.7–2.9)
AI/AN	0.4 (0.2–0.8)	0.8 (0.5–1.5)	0.5 (0.2–1.3)	0.9 (0.4–1.6)	1.3 (0.7–2.3)	1.3 (0.6–2.5)
White	0.5 (0.4–0.5)	0.4 (0.3–0.5)	0.4 (0.3–0.5)	1.2 (1.1–1.3)	1.0 (0.9–1.1)	0.9 (0.8–1.0)
Model 3: Additionally adjusts for AUDIT-C (0–12)						
Latine	0.2 (0.1–0.3)	0.2 (0.1–0.3)	0.2 (0.1–0.3)	0.6 (0.4–0.8)	0.5 (0.3–0.7)	0.5 (0.3–0.7)
Asian	0.1 (0.1–0.2)	0.0 (0.0–0.1)	0.1 (0.1–0.2)	0.3 (0.2–0.4)	0.4 (0.3–0.6)	0.3 (0.2–0.4)
Black	0.2 (0.1–0.3)	0.2 (0.1–0.3)	0.3 (0.2–0.5)	0.7 (0.5–0.9)	0.7 (0.5–1.0)	0.6 (0.3–0.9)
NH/PI	0.1 (0.1–0.3)	0.1 (0.0–0.3)	0.1 (0.0–0.6)	0.5 (0.3–1.0)	0.4 (0.2–0.9)	1.0 (0.5–2.0)
AI/AN	0.2 (0.1–0.5)	0.4 (0.2–0.8)	0.3 (0.1–0.6)	0.5 (0.3–1.0)	0.9 (0.5–1.6)	0.7 (0.3–1.4)
White	0.3 (0.2–0.3)	0.2 (0.2–0.2)	0.2 (0.2–0.2)	0.7 (0.6–0.8)	0.7 (0.6–0.7)	0.5 (0.5–0.6)

## Discussion

This study describes the prevalence of provider-documented AUD in primary care across subgroups based on the intersection of race, ethnicity, sex, and SES. Several patterns were observed across subgroups. First, the prevalence of AUD was very low in all racial or ethnic, sex, and SES groups compared to clinical estimates of AUD from prior studies in the VA not restricted to diagnoses in primary care (6.5%) and national estimates in the adult population (11.3%) [56, 67]. Second the prevalence of AUD appeared to vary across intersections of race, ethnicity, sex, and SES, but confidence intervals were wide and largely overlapping. Third, there were no consistent patterns across terciles of SES. Fourth, Asian patients had a lower prevalence compared to other racial and ethnic groups. Fifth, women had a lower prevalence compared to men. Lastly, patterns remained consistent despite adjustment for several factors expected to account for differences in provider-documented AUD, except for adjustment for alcohol use attenuating differences across some subgroups. Consistent with Intersectionality Theory, Fundamental Cause Theory, and Public Health Critical Race Praxis, these findings highlight how intersections of race, ethnicity, sex, and SES, and the upstream power structures that underpin them (racism, sexism, and social classism) result in differing prevalence of provider-documented AUD in primary care settings depending on one’s intersectional identity when providers have alcohol screening measures available to them.

The prevalence of provider-documented AUD in this study, ranging from 0.1 to 2.0% from Asian middle SES women to AI/AN high SES men, consistent with the 3.1% prevalence of documented AUD found in a prior non-VA study of Pacific Northwest United States primary care patients [28], was lower than in VA studies. For instance, a VA study reported a prevalence of provider-diagnosed AUD at 6.5% overall in women and men combined: 5.7% for White, 7.1% for Latine, and 9.8% for Black [67]. Differences in prevalence may be due to differences in the populations of patients who receive care in the VA and KPWA, lack of restriction of prior VA studies to primary care, or differences in the population and care setting. For example, VA patients are more often men (who have a higher prevalence of AUD than women) [67], have higher alcohol use compared to the general U.S. adult population [59], and are at increased risk of developing AUD in part due to disproportionate exposure to violence and trauma [66], (Dworkin et al., 2018). Additionally, the VA has accessible and affordable addiction services available to treat AUD [1, 61, 62, 65], and VA patients may be more willing to disclose drinking behavior and seek addiction services compared to other settings [67]. In contrast, during this study’s observation period, KPWA patients received specialty addiction treatment almost entirely outside the integrated delivery system via providers in the community [42] and could have reduced AUD diagnoses documented in the EHR in primary care. Lastly, given known underestimates of unhealthy alcohol use based on AUDIT-C screening in the VA [4], and the much higher prevalence of unhealthy alcohol use in the present study (Table 1), differences in the accuracy of AUDIT-C screening could contribute to different findings in the present study compared to VA studies.

The prevalence of AUD found in this study was also lower than in studies using “gold-standard” interview-based diagnostic measures, suggesting that AUD is underdiagnosed in primary care, consistent with a prior study that showed survey-based prevalence rates for AUD are higher than clinically documented AUD rates in VA [66]. For instance, the National Survey on Drug and Health (NSDUH) found a higher prevalence of AUD in U.S. adults in 2020: 11% overall, with a range from 4.8% for NH/PI, 7.9% for Asian, 10.1% for Latine, 10.8% for Black, 11.1% for White, and 14% for AI/AN adult respondents [54]. Differences in the observed prevalence of AUD found in NSDUH and KPWA may be due to several factors. NSDUH used detailed, semi-structured interviews that assessed 11 symptoms of AUD according to the AUD diagnosing criteria in DSM-5 [55]. AUD diagnoses in a clinical setting were largely dependent on whether and how providers assessed AUD, and whether patients were comfortable or willing to disclose AUD symptoms [32]. Typically, unstructured approaches to recognizing and diagnosing AUD in clinical settings may be more susceptible to bias, such as racism, sexism, and classism, given the stigma of AUD, and may be particularly biased for groups with intersecting lived experiences of stigma and discrimination resulting from systems of power and oppression [21, 38].

We anticipated that patients may be more likely to have provider-documented AUD if they lived in communities with lower SES, given people who live in disadvantaged communities are more likely to drink at higher levels [15]. Further individuals from low SES neighborhoods are less likely to seek treatment for AUD due to cost and access [36], potentially increasing the time between AUD onset and seeking treatment [23, 24] which may result in a higher likelihood of receiving a provider-documented AUD due to having greater AUD symptom severity at the time of diagnosis. However, this pattern across SES was only observed in White men, who made up 76% of the men in the sample, in whom lower SES tended to be associated with a higher prevalence of provider-documented AUD (1.8% in low SES, 1.7% in middle SES, and 1.5% in high SES). The unexpected inconsistent patterns across terciles of community-level SES within race or ethnic subgroups might be due to inadequate sample sizes of minoritized racial and ethnic groups. Future studies with larger sample sizes for minoritized patients are needed to detect SES-related differences with greater precision. Further, the observation of inconsistent patterns across terciles of SES found in this study is consistent with previous scientific literature [35]. Although some prior studies show that people living in higher SES areas engage in more frequent and heavier drinking [11], which may result in a higher prevalence of AUD, medical providers may not document stigmatized conditions, such as AUD, as often in those individuals [6].

In this study, Asian patients tended to have a lower prevalence of provider-documented AUD compared to any other race or ethnic group. This is consistent with a previous U.S. population-based study [22] that showed the prevalence of AUD was generally lower among Asian respondents. However, although the prevalence of AUD diagnosis among Asian Americans is generally lower, Asians who have AUD appear to have disproportionately lower rates of alcohol treatment utilization [26].

Also consistent with patterns of AUD observed in the U.S. general population, White patients in this study had a higher prevalence compared to Black patients [54]. We expected similar results as previous VA studies evaluating AUD in clinical settings, which found that Black patients had a higher prevalence of AUD documented in the EHR compared to White patients, even after adjusting for patient-reported alcohol consumption [63, 66] given the unstructured nature of provider-documented AUD. Consistent with potential racial bias found in VA studies, prior studies found that Black patients were more likely than White patients to have stigmatizing language found in hospital notes within EHRs [29, 58]. Therefore, we theorized that bias in AUD diagnosing patterns in clinical settings likely stems, at least in part, from clinicians’ implicit and explicit bias formed by the societal context characterized by pervasive structural racism. For instance, clinicians have historically been taught that Black patients had a higher pain tolerance than White patients, leading to the administration of lower doses of pain medication to Black patients [30]. The contradictory results found in this study may be due KPWA’s comprehensive alcohol screening process, which despite not capturing the true prevalence of AUD, may have decreased provider-documented AUD diagnosis bias in Black compared to White patients. Another potential explanation could be that Black people, due to upstream factors that restrict access to care due to racism, are less likely to have had the chance to be diagnosed in primary care settings in the first place.

The prevalence of provider-documented AUD was higher among men compared to women, consistent with previous clinical studies among veterans [63, 66, 67] and in the U.S. general population [22, 54]. This pattern remained despite adjustments for alcohol use via AUDIT-C, suggesting that even after adjusting for higher reported alcohol use by men, men were still diagnosed with AUD at a higher rate than women. Of note, despite women being diagnosed with AUD at lower rates compared to men, prior studies suggest women experience a disproportionately greater burden from alcohol use [46] and stigma compared to men [37].

### Limitations

The use of EHR data from a large sample of 439,375 primary care patients made it feasible to study provider-documented AUD across intersections of race or ethnicity, sex, and SES. However, relying on EHR data presented several limitations. For instance, EHR-ascertained race, ethnicity, sex, and community-level SES cannot fully capture the multiple factors that influence how much a person is exposed to or experiences structural discrimination associated with intersecting identities. This may influence levels of alcohol consumption and/or the likelihood of receiving a provider-documented AUD diagnosis. Furthermore, the provider-documented AUD in this study was limited to diagnoses made by primary care clinicians in KPWA’s EHR. Excluding external claims data for AUD diagnoses provided by clinicians in the community may have reduced the prevalence of provider-documented AUD observed in this study and may have under-estimated AUD in patients uncomfortable disclosing symptoms of AUD when they are in the EHR, especially patients experiencing racism, and socioeconomic disadvantages. Moreover, our sample was predominantly White, with smaller sample sizes for minoritized race and ethnic groups. This study was conducted in a single integrated health system in Washington State; findings may not generalize to other clinical settings. Additionally, this study did not measure the true prevalence of AUD in KPWA patient subgroups. While we expected the prevalence of AUD documented in the EHR to typically underestimate the true prevalence due to provider under-recognition, in some instance, providers may over-diagnose AUD.

The study also has important strengths. This study contributed to the scientific literature by exploring provider-documented AUD by race, ethnicity, sex, and SES in a non-veteran primary care setting in a regionally integrated healthcare system caring for large numbers of women and men that has implemented high-quality patient self-report screening, allowing assessment of the prevalence of documented AUD after adjusting for alcohol use. The outcome reflects AUD recognized and documented in primary care. The study had access to patient addresses that were used to assess community-level SES. Important covariates were used in stepwise regression modeling, allowing for stepped adjustment of multiple important factors that could have impacted the interpretation of findings. Finally, this study provides support for why increasing AUD diagnosing in primary care is necessary for increasing access to evidenced-based AUD treatments available in clinical settings.

## Conclusions

This study contributes to the understanding of how patients’ intersecting identities may be reflected in the documentation of AUD diagnoses in medical settings. The prevalence of provider-documented AUD varied across intersections of race, ethnicity, sex, and SES. Unlike prior population-based studies using EHR data in VA, except for AI/AN patients, minoritized race or ethnic groups had a lower prevalence of AUD than White patients across sex and a spectrum of SES. There were no consistent patterns across terciles of SES within subgroups of intersections based on race, ethnicity, and sex. Further research using larger samples of minoritized groups across a spectrum of SES is needed to understand differences in provider-documented AUD across intersections of race, ethnicity, sex, and SES.

### Supplementary Information

Supplementary Material 1

## Data Availability

Data are not publicly available due to institutional rules regarding data sharing.

## Abbreviations

AUD: Alcohol use disorder
UAU: Unhealthy alcohol use
AUDIT-C: Alcohol use disorders identification tests-consumption
SES: Community-level socioeconomic status
MNDI: Messer neighborhood deprivation index
KPWA: Kaiser Permanente Washington
VA: Veterans Health Administration
