# Supplementary material for: Prevalence of alcohol use disorders documented in electronic health records in primary care across intersections of race or ethnicity, sex, and socioeconomic status
Source: Addict Sci Clin Pract. 2024 Aug 30;19:61. doi: 10.1186/s13722-024-00490-6 (PMC11365182; doi:10.1186/s13722-024-00490-6)
Supplement: Supplementary file 1 — Supplementary Material 1 [file 13722_2024_490_MOESM1_ESM.docx]

**SUPPLEMENT**

**Supplemental Figure 1.** Conceptual model used in this study to describe patterns of clinically-documented AUD across intersections of race or ethnicity, sex, and terciles of community-level SES.

Factors depicted in **bold** are those that could be measured using this study’s dataset.

**Supplemental Figure 2.** Alcohol Use Disorders Identification Test-Consumption (AUDIT-C)

**Supplemental Table 1.** Messer Neighborhood Deprivation Index (MNDI)

**Supplemental Table 2**. ICD-9 and ICD-10 Active AUD Diagnosis Codes

**Supplemental Table 3.** Detailed list of alcohol-related medical conditions and mental health diagnoses.


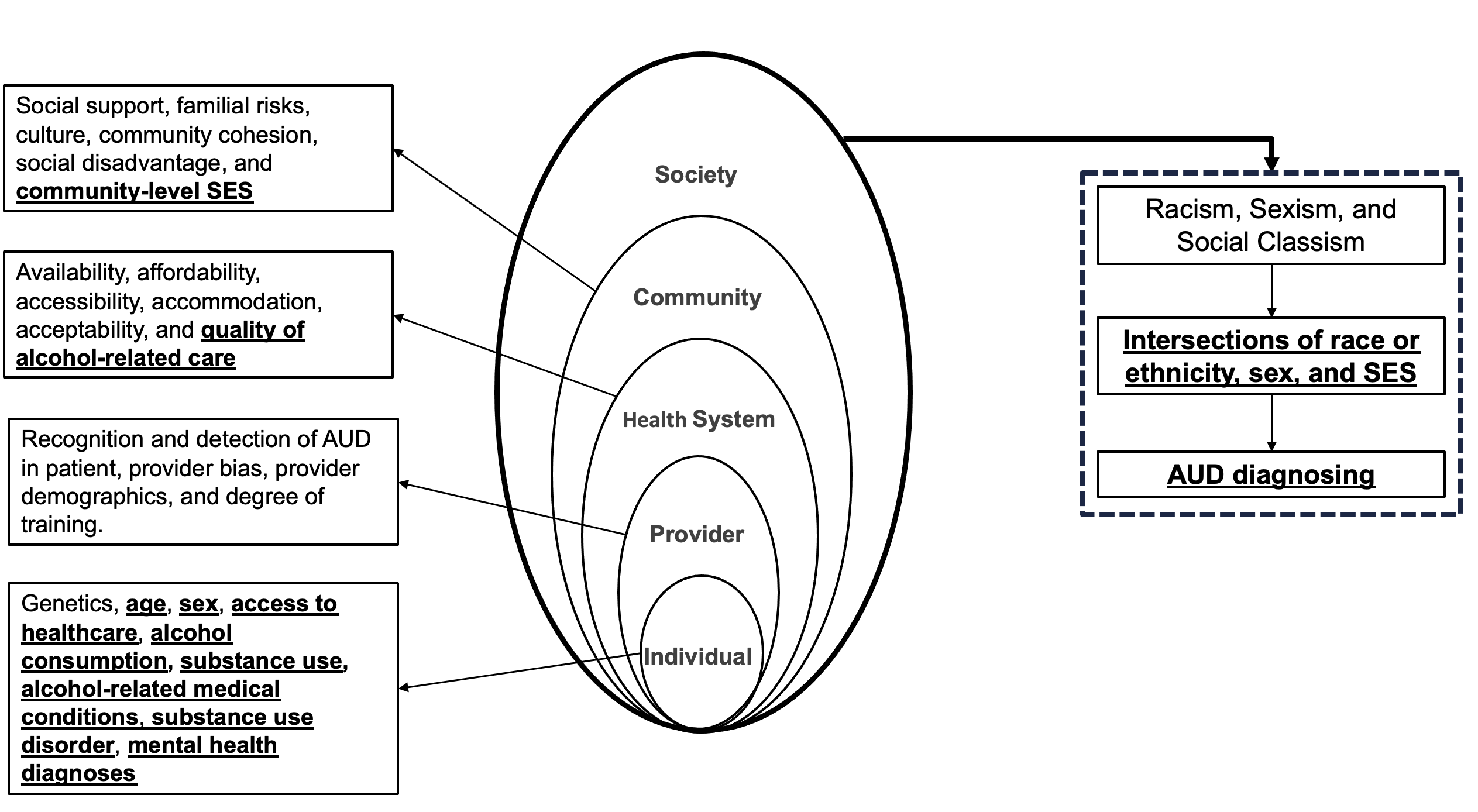


**Supplemental Figure 1 (above).** Conceptual model used in this study to describe patterns of clinically-documented AUD across intersections of race or ethnicity, sex, and terciles of community-level SES.

Factors depicted in **bold** are those that could be measured using this study’s dataset.

**Supplemental Figure 2.** The text used for the AUDIT-C (Alcohol Use Disorders Identification Test-Consumption version)


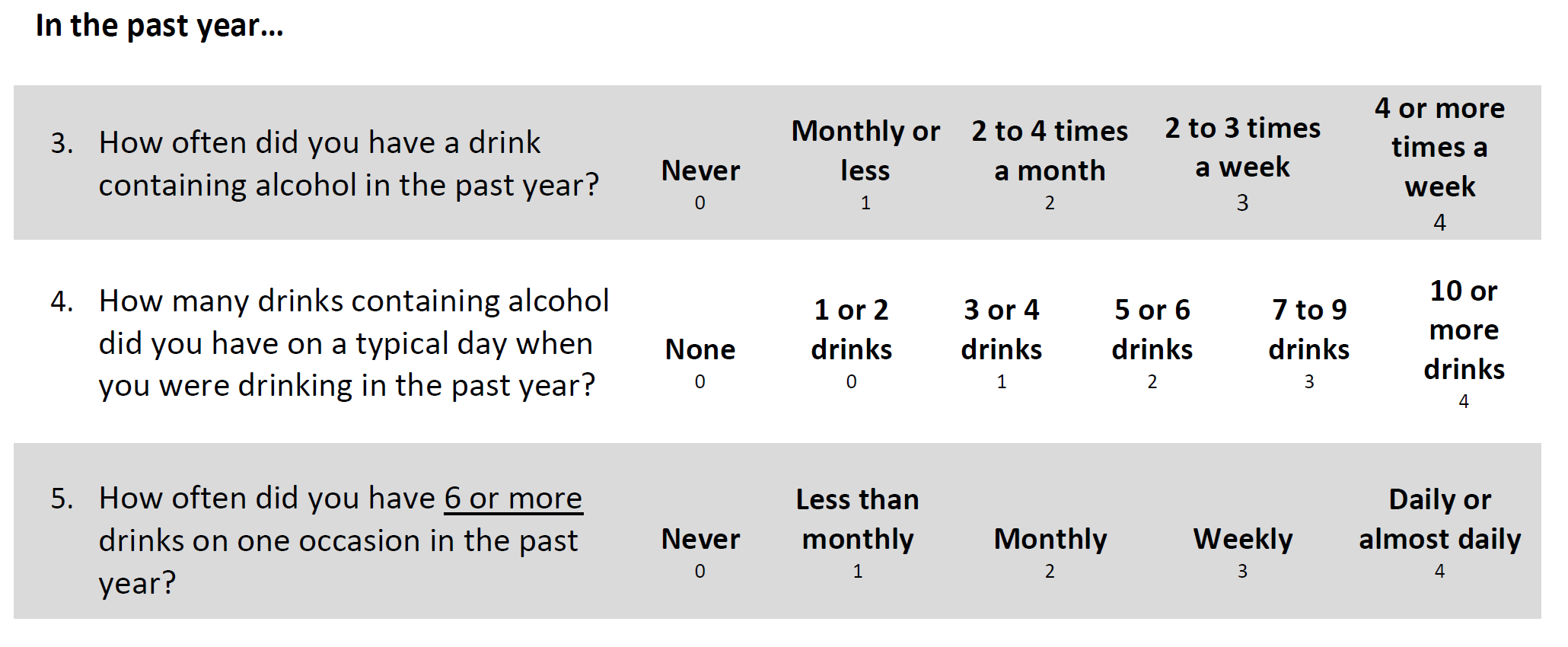


Alcohol Use Disorders Identification Test-Consumption (AUDIT-C) is a 3-item self-report screening questionnaire that measures the typical quantity and frequency of alcohol consumption and heavy episodic drinking. Items are answered on a 5-point scale (0–4 points) and then summed (total score 0–12 points). The AUDIT-C reflects average drinks per day with exponential increases above scores of 6 and increasing scores are associated with an increased likelihood of AUD (Rubinsky et al., 2013)

**Supplemental Table 1.** Messer Neighborhood Deprivation Index (MNDI)

| **8 Variables that make up Messer Neighborhood Deprivation Index (MNDI)** |
| --- |
| 1) % of the adult population with less than a high school diploma |
| 2) % of households earning less than $30,000 per year |
| 3) % of households with below-poverty level income |
| 4) proportion of the civilian non-institutionalized population between 18 and 64 who are unemployed |
| 5) proportion of households on public assistance |
| 6) % living in crowded housing |
| 7) proportion of households headed by females (no male present) with dependent children |
| 8) % of males in management or professional occupations |

Principle component analysis (PCA) was used to standardize MDNI scores and to determine the weight of each variable (Messer et al., 2006)

**Supplemental Table 2**. ICD-9 and ICD-10 Active AUD Diagnosis Codes

| **ICD-9 and ICD-10 AUD Diagnosis Codes** |
| --- |
| 291.2, 291.81, 291.89, 291.9, 303.90, 303.91, 303.92, 305.00, 305.01, 305.02, F10.10, F10.120, F10.121, F10.129, F10.13, F10.130, F10.131, F10.132, F10.139, F10.14, F10.15, F10.150, F10.151, F10.159, F10.18, F10.180, F10.181, F10.182, F10.188, F10.19, F10.20, F10.220, F10.221, F10.229, F10.230, F10.231, F10.232, F10.239, F10.24, F10.250, F10.251, F10.259, F10.26, F10.27, F10.280, F10.281, F10.282, F10.288, F10.29 |

**Supplemental Table 3.** Detailed list of alcohol-related medical conditions and mental health diagnoses.

| **Alcohol-related medical conditions:** |
| --- |
| alcoholic psychosis, polyneuropathy, alcohol cardiomyopathy, gastritis, liver disease, acute and chronic pancreatitis, portal hypertension, esophageal varices, gastroesophageal hemorrhage, degeneration of nervous system due to alcohol, alcohol myopathy, and liver cirrhosis unspecified |
| **Mental health diagnoses:** |
| anxiety disorders, attention deficit hyperactivity disorder (ADHD), bipolar disorder, depressive disorder, eating disorder, psychosis, post-traumatic stress disorder (PTSD), and schizophrenia |
